# Time dynamics of the *Bacillus cereus* exoproteome are shaped by cellular oxidation

**DOI:** 10.3389/fmicb.2015.00342

**Published:** 2015-04-22

**Authors:** Jean-Paul Madeira, Béatrice Alpha-Bazin, Jean Armengaud, Catherine Duport

**Affiliations:** ^1^UMR408, Sécurité et Qualité des Produits d'Origine Végétale, Université d'AvignonAvignon, France; ^2^INRA, UMR408, Sécurité et Qualité des Produits d' Origine VégétaleAvignon, France; ^3^Commissariat à l'énergie Atomique et aux Énergies Alternatives (CEA), Direction des Sciences du Vivant (DSV), IBEB, Li2DBagnols sur Cèze, France

**Keywords:** exoproteome, *Bacillus cereus*, shotgun proteomics, methionine oxidation, toxins

## Abstract

At low density, *Bacillus cereus* cells release a large variety of proteins into the extracellular medium when cultivated in pH-regulated, glucose-containing minimal medium, either in the presence or absence of oxygen. The majority of these exoproteins are putative virulence factors, including toxin-related proteins. Here, *B. cereus* exoproteome time courses were monitored by nanoLC-MS/MS under low-oxidoreduction potential (ORP) anaerobiosis, high-ORP anaerobiosis, and aerobiosis, with a specific focus on oxidative-induced post-translational modifications of methionine residues. Principal component analysis (PCA) of the exoproteome dynamics indicated that toxin-related proteins were the most representative of the exoproteome changes, both in terms of protein abundance and their methionine sulfoxide (Met(O)) content. PCA also revealed an interesting interconnection between toxin-, metabolism-, and oxidative stress–related proteins, suggesting that the abundance level of toxin-related proteins, and their Met(O) content in the *B. cereus* exoproteome, reflected the cellular oxidation under both aerobiosis and anaerobiosis.

## Introduction

The gram-positive, motile bacterium, *Bacillus cereus*, is a well-known agent of gastrointestinal (GI) tract infection (Stenfors Arnesen et al., 2008; Bishop et al., 2010). The critical step of infection occurs in the small intestine, where *B. cereus* encounters carbohydrate starvation conditions and changing oxygenation and oxidoreduction potential (ORP) conditions (Guyton, 1977; Moriarty-Craige and Jones, 2004; Fabich et al., 2008; Marteyn et al., 2010). During the course of infection, the survival and growth of *B. cereus* depend on the secretion and release into the extracellular compartment of multiple proteins (Gilois et al., 2007; Gohar et al., 2008). The *B. cereus* ATCC 14579 exoproteome, which comprises the secreted proteins and all the other released proteins found in the pathogen's extracellular surroundings (Armengaud et al., 2012), was recently established for cells grown under conditions considered to mimic those encountered in the human intestine, i.e., low-ORP anoxic conditions, high-ORP anoxic conditions, and oxic conditions, in pH-regulated culture using glucose as the sole carbohydrate source (Clair et al., 2010). The *B. cereus* exoproteome is dominated by toxin-related proteins (~35% of the exoproteome, as estimated by spectral count) and degradative enzymes plus adhesins (~35% of the exoproteome), which are all recognized as major virulence factors (Stenfors Arnesen et al., 2008; Ingmer and Brondsted, 2009; Kamar et al., 2013; Ramarao and Sanchis, 2013). The other components of the *B. cereus* exoproteome comprise components of the flagellar apparatus (~15% of the exoproteome), as well as an important number of proteins that lack export signal sequences, accounting for 15% of the exoproteome. These proteins, found more abundantly in the cytoplasm, include metabolic enzymes (mainly glycolytic enzymes), translation-related proteins, molecular chaperones, and antioxidant enzymes such as catalase, hydroperoxide reductase, and superoxide dismutase. Several studies have reported the moonlighting activities of these proteins, which are involved in bacterial virulence. Most enzymes in the glycolytic pathway, tricarboxylic acid (TCA) cycle and glyoxylate cycle have adhesive properties that aid in interacting with the host extracellular matrix. The most common moonlighting activity of bacterial molecular chaperones is to activate (or inhibit) mononuclear phagocyte cytokine synthesis. Antioxidants produced by *Mycobacterium bovis* suppress host immune response (Sadagopal et al., 2009; Vellasamy et al., 2009; Henderson and Martin, 2011).

*B. cereus* adjusts its primary metabolism to grow efficiently under aerobic respiratory and anaerobic fermentative conditions and to adapt to low-ORP conditions (Duport et al., 2006; Clair et al., 2012). In addition, as for all other bacteria, *B. cereus* undergoes a major metabolic switch from primary metabolism (exponential growth) to secondary metabolism (stationary phase) in response to nutrient starvation or oxidative stress (Nieselt et al., 2010). Aerobic respiration relies on dioxygen to drive ATP production via the respiratory chain (Duport et al., 2006). One caveat is that this process is accompanied by a major production of reactive oxygen species (ROS) (Gonzalez-Flecha and Demple, 1995; Brynildsen et al., 2013; Imlay, 2013). In addition to the respiratory chain, endogenous ROS can be generated in response to starvation (nutrient stress) as a secondary stress (Mols and Abee, 2011). Under anaerobiosis, *B. cereus* catabolizes glucose-using, fermentative pathways, which are not recognized as high-ROS-producing pathways under normal conditions. However, low-ORP conditions can induce ROS production in response to reductive stress (Clair et al., 2012). Bacteria use a large spectrum of ROS scavenging systems, including low-molecular-weight molecules, metabolites, and antioxidant enzymes, to maintain ROS at non-toxic levels and to prevent macromolecule damage (Chi et al., 2011; Mailloux et al., 2011). Amino acid residues in proteins represent one of the major targets of ROS and cellular oxidants. The two amino acids that are the most prone to oxidative attack by ROS are cysteine and methionine (Met), both of which contain susceptible sulfur atoms. However, Met residues are the most susceptible to oxidation by almost all forms of ROS (Vogt, 1995; Stadtman et al., 2005). Met oxidation produces a stable product, methionine sulfoxide, Met(O), which can be detected readily by mass spectrometry through a mass increase of 15.9949 atomic mass units. Thus, Met oxidation might serve as a sensitive marker for proteins oxidized by ROS.

The objective of the present study was to define the exoproteome time dynamics of *B. cereus* grown in three ORP conditions, and to assess by tandem mass spectrometry the oxidation level of the secreted proteins, which should be correlated with the cellular oxidation level. For this purpose, we collected *B. cereus* supernatant at three points of the time-growth curve, i.e., during early exponential growth phase (EE), at the late exponential growth phase (LE) signifying the transition between exponential and stationary phases, and during the stationary phase (S). This was performed for cells grown under aerobiosis, as well as under high- and low-ORP anaerobiosis. Time-course changes in terms of exoprotein abundance level and the Met(O) peptide content of exoproteins were assessed by high-throughput nanoLC-MS/MS (Clair et al., 2010). The repertoire of experimentally confirmed exoproteins of *B. cereus* presented here is the largest ever reported, and more interestingly provides new insights into the interplay between toxin-related protein secretion and intracellular ROS production.

## Materials and methods

### *B. cereus* growth conditions

*B. cereus* ATCC 14579 cells were grown in a batch bioreactor on MOD medium supplemented with 30 mM glucose as the carbon source (Rosenfeld et al., 2005) and buffered at pH 7.2 with 2 M KOH. The bioreactor was an autoclavable 3-liter glass BioFlo®/CelliGen®115 (New Brunswick Scientific) with a working volume of 2 liters. It was equipped with a polarographic oxygen electrode (Mettler Toledo), a pH electrode (Mettler Toledo), and a redox-combined electrode (AgCl, Mettler Toledo). Sterile gas was fed through the culture at a constant flow set to 20 mL/h. For oxic conditions, oxygen saturation was maintained at 100% by automatic adjustment of the stirring speed. For anoxic conditions, a dissolved oxygen tension value (*p*O_2_) of 0% was obtained with a constant flow of pure nitrogen (high- ORP condition) or hydrogen gas (low-ORP condition). Each bioreactor was inoculated with a subculture grown for 8 h (exponential growth phase) in glucose-containing MOD medium under aerobiosis or anaerobiosis. Cells from the inocula were harvested by centrifugation (7000 × g for 5 min at room temperature), washed in fresh medium, and then diluted to achieve an initial optical culture density at 600 nm of 0.02. Batch cultures were carried out at 37°C under a 300 rpm agitation speed.

### Exoproteome preparations and trypsin in-gel proteolysis

For each of the three growth conditions, three independent growth cultures in a fermenter were carried out, resulting in biological samples in triplicate for each time point. Optical density, ORP, and *p*O_2_ were monitored every 30 min during the bacterial growth. The growth rate was determined from the absorbance data. A 200-mL sample of the culture was systematically taken at the exponential, transition, and stationary phases for the nine bioreactor cultures. Cell pellets and extracellular media were separated by centrifugation at 10,000 × g for 10 min at 4°C. The extracellular media were successively filtered through acetate membrane filters (Sartorius) with pore sizes of 0.85, 0.45, and 0.20 μm, respectively. Proteins from the 27 samples were precipitated by adding 10 mL trichloroacetic acid solution at 100% (w/v) to 40 mL filtered solution. The precipitated material was recovered after overnight incubation at 4°C by centrifugation at 7000 × g for 15 min at 4°C, and the extracellular proteins in the resulting pellet were then dissolved in 100 μ L NUPAGE® LDS (Lithium dodecyl sulfate) sample buffer 1X (Invitrogen) supplemented with β-mercaptoethanol. Samples were boiled for 5 min at 95°C, sonicated for 5 × 5 s in a transonic 780H sonicator and loaded on NuPAGE® Novex 4–12% Bis-Tris gels (Invitrogen) that were run for a short 5-min migration at 200 V using NuPAGE® MES supplemented with NuPAGEantioxidant as the running buffer (Hartmann and Armengaud, 2014). This avoids any artifactual protein oxidation. Gels were stained with Simply Blue SafeStain, a ready-to-use Coomassie G-250 stain from Invitrogen. After overnight destaining, the single band of each gel lane was cut and divided into 2 fractions, each corresponding to a 3 × 4 mm^2^ polyacrylamide band. The 54 resulting polyacrylamide gel pieces were processed for further destaining, reduction and iodoacetamide treatments, and in-gel proteolysis with trypsin (Roche) in the presence of ProteaseMax additive (Promega), as previously described (De Groot et al., 2009; Clair et al., 2010). The two digests obtained from the same sample were pooled as a single peptide mixture. Exponential phase samples were injected without being diluted, due to their lower protein content, while the samples collected at the transition and stationary phases were diluted 1:50 in 0.1% trifluoroacetic acid prior to nanoLC-MS/MS analysis.

### Tandem mass spectrometry

NanoLC-MS/MS experiments were performed using an LTQ-Orbitrap XL hybrid mass spectrometer (ThermoFisher) coupled to an UltiMate 3000 nRSLC system (Dionex ThermoFisher), in similar conditions to those previously described (Dedieu et al., 2011). Peptide mixtures were loaded and desalted on-line on a reverse-phase precolumn (Acclaim PepMap 100 C18, 5 μm bead size, 100 Å pore size, 300 μm i.d × 5 mm (Dionex-ThermoFisher). Peptides were then resolved on a Dionex nanoscale Acclaim Pepmap100 C18 capillary column (3 μm bead size, 100 Å pore size, 75 μm i.d. × 15 cm) at a flow rate of 0.3 μ L/min using a 90 min. gradient from 4 to 40% solvent B (0.1% HCOOH/100% CH_3_CN) prior to injection into the mass spectrometer. Solvent A was 0.1% HCOOH/100% H_2_O. Full-scan mass spectra were measured from *m/z* 300 to 1800 with the LTQ-Orbitrap XL mass spectrometer in data-dependent mode using TOP3 strategy. In brief, a scan cycle was initiated with a full scan of high mass accuracy in the Orbitrap, followed by MS/MS scans in the linear ion trap on the three most abundant precursor ions, with 60 s dynamic exclusion of previously selected ions.

### Protein identification

Peak lists from the tandem mass spectrometry raw data were generated with the MASCOT DAEMON software (version 2.3.2) from Matrix Science using the extract_msn.exe data import filter from the Xcalibur FT package (version 2.0.7) proposed by ThermoFisher. Data import filter options were set as follows: at 400 (minimum mass), 5000 (maximum mass), 0 (grouping tolerance), 0 (intermediate scans), and 1000 (threshold). Using the MASCOT search engine (version 2.3.02) from Matrix Science, we searched all MS/MS spectra against an in-house polypeptide sequence database containing the sequences of all annotated proteins encoded by the *B. cereus* ATCC 14579 chromosome (NC_004722) and plasmid, pBClin15 (NC_004721), supplemented with 44 new proteins discovered by a previous proteogenomic analysis (unpublished data). This database comprises 5299 polypeptide sequences, totaling 1,464,675 amino acids. Searches for tryptic peptides were performed with the following parameters: full trypsin specificity, a mass tolerance of 5 ppm on the parent ion and 0.6 Da on the MS/MS, static modifications of carboxyamidomethylated Cys (+57.0215), and dynamic modifications of oxidized Met (+15.9949). The maximum number of missed cleavages was set at 2. All peptide matches with a peptide score below a *p*-value of 0.05 were parsed using the IRMa 1.28.0 software (Dupierris et al., 2009). A protein was considered to be validated when at least two different peptides were detected in the same sample. The false-positive rate for protein identification was estimated using the appropriate decoy database as below 0.1% with these parameters.

### Label-free protein quantification and statistical analysis

The number of MS/MS spectra per protein (spectral counts) was extracted for the 27 samples and used for protein quantitation. The normalized spectral abundance factor (NSAF) was calculated by dividing the spectral count for each observed protein by the polypeptide theoretical mass, as described previously (Christie-Oleza et al., 2012). Principal component analysis (PCA) was carried out with R version 3.0.1 (http://cran.r-project.org/bin/windows/base/old/3.0.1/). The data analyses were performed with “FactoMineR,” a package written in R dedicated to multivariate exploration data analysis (Lê et al., 2008). PCA was carried out with biological replicates of each growth phase as individuals and the spectral counts of proteins as quantitative variables. The correlation coefficients between the variable and the coordinates of the individuals on the axis were calculated for all the variables, dimension by dimension. The significance of each correlation coefficient was calculated using a Student's *t*-test. Variables, for which the *p*-value associated with this test was smaller than 0.05, are reported in Table S4 in Supplementary Material.

### Proteomic data repository

The mass spectrometry proteomics data have been deposited in the ProteomeXchange Consortium (http://proteomecentral.proteomexchange.org) via the PRIDE partner repository (http://www.ebi.ac.uk/pride/), with the dataset identifier PXD001482 and DOI 10.6019/PXD001482.

## Results and discussion

### Comparative exoproteome, large survey

#### Growth kinetics of *B. cereus* ATCC 14579

**Figure 1:**
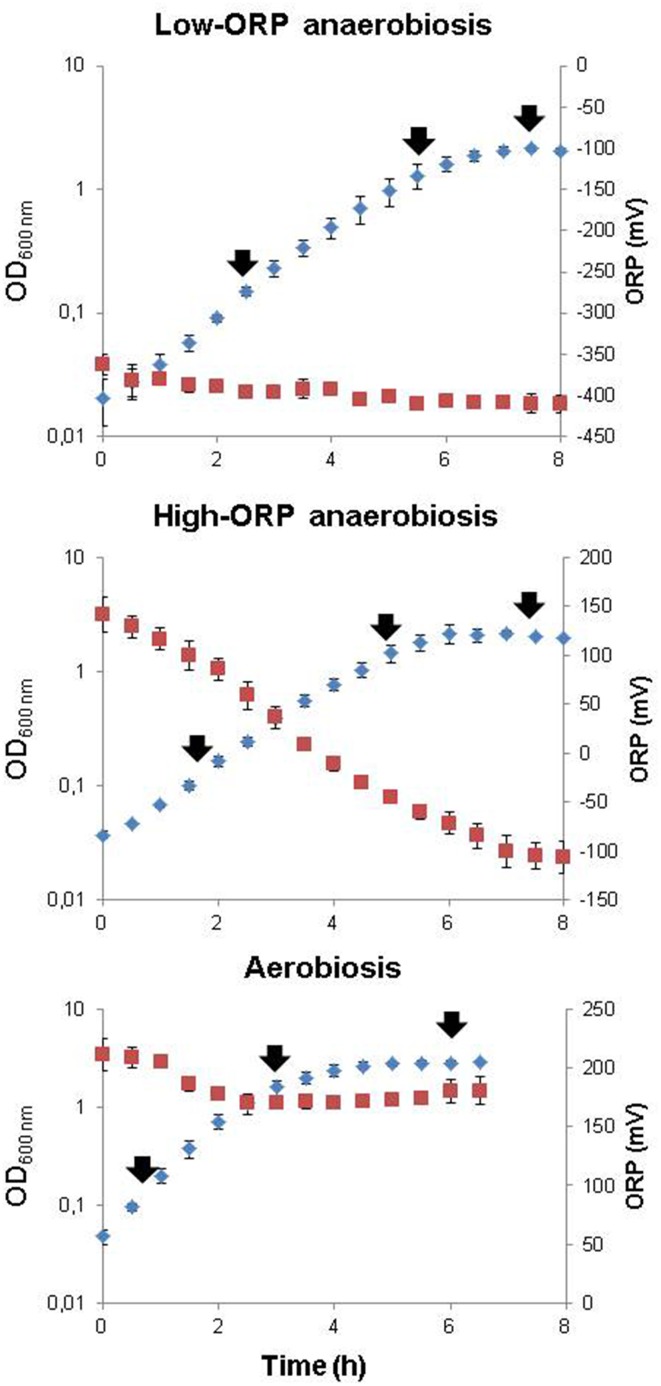
**Growth curves of *B. cereus* in pH-regulated batch culture under aerobiosis, high-ORP anaerobiosis, and low-ORP anaerobiosis**. The results from biological triplicate curves are indicated. Optical densities (OD_600 nm_) and ORP values are shown in blue and red, respectively. Samples for exoproteomic analyses were taken during the exponential growth phase (EE), late growth phase (LE), and stationary growth phase (S), as indicated by black arrows.

Bacteria were grown in pH- and temperature-regulated bioreactors using glucose as the sole carbon source (pH 7, 37°C, 30 mM glucose). Growth was investigated under aerobiosis (pO_2_ = 100%) and anaerobiosis (pO_2_ = 0%). Two different ORP conditions were obtained under anaerobiosis: a high-ORP anoxic condition (initial ORP = 130 ± 20 mV) and a low-ORP anoxic condition (iORP = −390 ± 35 mV), this latter condition being achieved under flux of hydrogen, a non-toxic reducing agent. Three biological replicates were performed per culture condition. Figure 1 shows the *B. cereus* growth curves and the extracellular ORP profiles established for the three culture conditions. As reported previously (Clair et al., 2012), *B. cereus* cells grew more slowly and produced less biomass in anoxic fermentative conditions than in oxic respiratory conditions. Changes in the initial extracellular ORP did not alter the growth rate and biomass production under fermentative anoxic conditions (Table S1 in Supplementary Material). However, the extracellular ORP profile differed significantly in the three conditions. Under aerobiosis (initial ORP = 210 ± 13 mV), the ORP dropped rapidly to its minimal value (final ORP = 184 ± 11 mV). This reflects the rapid consumption of dissolved oxygen through respiration, to generate ATP for growth (Rosenfeld et al., 2005). The ORP measured under high-ORP anoxic fermentative conditions (iORP = 130 ± 20 mV) decreased concomitantly with the biomass increase to a reach a minimal value of −106 ± 16 mV, while under low-ORP conditions the ORP remained constant (iORP = −390 ± 35 mV and fORP = −410 ± 10 mV). Clearly, the reducing capacity of *B. cereus* cells is higher under high-ORP anaerobiosis than under low-ORP anaerobiosis (Le Lay et al., 2015). To examine the changes in exoproteome profiles associated with growth, samples were taken at the time points indicated by the arrows in Figure 1, i.e., during early exponential growth phase (EE), late exponential growth phase (LE), and stationary phase (S). Proteins from the 27 filtered supernatants were concentrated by precipitation with trichloroacetic acid. The resulting samples were then dissolved into NuPAGE LDS sample buffer supplemented with β-mercaptoethanol to prevent protein oxidation. Samples were loaded on NUPAGE® precast gels that were run for a short migration time only (Hartmann and Armengaud, 2014). NUPAGE® antioxidant was added in the upper buffer chamber to maintain the reduced state of the proteins during the run and avoid any protein oxidation. Each sample was excised from the gel as a polyacrylamide band. Trypsin proteolysis was carried out *in*-gel. The resulting peptides were analyzed by shotgun tandem mass spectrometry (Clair et al., 2010). A total of 120,470 MS/MS spectra were detected when considering the three biological repeats. Among them, 50,828 were assigned to *B. cereus* peptide sequences (Table S2 in Supplementary Material). A total of 392 proteins were identified based on the confident detection of at least two different peptides (Table S3 in Supplementary Material).

#### New mass spectrometry–identified exoproteins

Compared to previous large shotgun proteomic studies on exoproteomes from *B. cereus* ATCC 14579 (Clair et al., 2010; Laouami et al., 2014), a total of 32 proteins were detected for the first time. These 32 new mass spectrometry–certified proteins account for 11% of the exoproteome, as assessed by the global sum of their normalized spectral count abundance factors (NSAF) cumulated over the 27 samples (Table S3 in Supplementary Material). Table 1 shows the sequence similarity–based functional annotation of these proteins and their abundances under aerobiosis, high-ORP- and low-ORP anaerobiosis. The 32 proteins could be categorized into three groups. Group A comprises 11 proteins that were not annotated in the first annotation report of the genome (Ivanova et al., 2003), but have been indicated by a proteogenomic study (unpublished data). Group B comprises 9 proteins that did not accumulate in EE growth phase in all the conditions tested, which explains why they were not detected in our previous study focused on this growth stage (Clair et al., 2010; Laouami et al., 2014). The protocol used in the present study probably favored the detection of the 12 other proteins (group C), which were found in very poor abundance. Among the new proteins identified, we identified a protein exhibiting high sequence similarity with the three putative enterotoxins, EntA, EntB, and EntC (Clair et al., 2010), and that we named EntD (unpublished results). Like EntD, 13 proteins comprised a predicted peptide signal. These were classified into cell-wall/cell-surface biogenesis, degradation/adhesion, and transport functional groups on the basis of data available in the literature and/or using the information available in the Kegg classification (Table S3 in Supplementary Material). The other proteins did not contain typical peptide signals and were classified as flagella components (BC1641 and BC1642), enzymes of the central glycolytic pathway (TpiA-BC5137 and Pgk-BC5138), enzymes of amino acid–related metabolic pathways (ArgC and GlnA), chaperones (BC1161-PrsA2), translation/transcription-associated proteins (BC1177), and proteins with unknown functions (BC4122 and BC1649).

**Table 1:** **Novel exoproteins identified in this study**.

**Gene**	**Accession n° (NP)**	**Protein name**	**Functionnal annotation**	**Secretion signals and functional domains^a^**	**Total NSAF^b^**								
					**Low-ORP anaerobiosis**			**High-ORP anaerobiosis**			**Aerobiosis**		
					**EE**	**LE**	**S**	**EE**	**LE**	**S**	**EE**	**LE**	**S**
**GROUP** **A^c^**													
BC1177	NA		Tryptophanyl-tRNA synthetase		0.00	0.00	**0.19**	0.00	0.00	**0.14**	0.00	0.00	**0.27**
NA	NA	ArgC	Gamma-glutamylphosphate reductase		**0.06**	0.00	**0.06**	0.00	0.00	**0.03**	**0.03**	0.00	**0.09**
BC3716	NA	EntD	Enterotoxin-like	S, SH3	**0.19**	**0.15**	**0.19**	**0.65**	**0.27**	**0.23**	**0.19**	**0.27**	**0.34**
BC4122	NA		2′, 3′-cyclic nucleotide 2′-phosphodiesterase		**0.09**	**0.05**	**0.02**	**0.23**	**0.08**	**0.05**	**0.20**	**0.01**	0.00
BC5138	NA	Pgk	Phosphoglycerate kinase		**0.90**	**0.66**	**1.74**	**0.49**	**1.15**	**2.00**	**0.40**	**0.07**	**1.25**
BC5137	NA	TpiA	Triosephosphate isomerase,		**1.36**	**0.94**	**2.87**	**1.36**	**1.25**	**2.98**	**0.42**	**0.23**	**1.32**
BC1649	NA		Unknown		**0.02**	0.00	**0.02**	0.00	0.00	0.00	**0.46**	**0.02**	0.00
NA	NA	CnaE	Collagen adhesion protein	S	**0.95**	**0.44**	**0.51**	**1.27**	**0.76**	**0.32**	**0.70**	**0.76**	**0.44**
NA	NA	SipB	Signalpeptidase	S	**0.66**	**0.53**	**0.79**	**0.99**	**0.92**	**0.79**	**0.73**	**0.92**	**1.25**
BC3705	NA	GlnA	Glutamine synthetase		**1.00**	**0.30**	**0.74**	**0.48**	**0.40**	**0.78**	**0.78**	**0.38**	**1.20**
BC3763	NA		Cellwallhydrolase	S	0.00	**1.55**	**1.59**	**0.17**	0.00	0.00	**0.97**	**0.55**	0.00
**GROUP** **B**													
BC0602	NP_830419	NprA	Neutralprotease	S	0.00	0.00	0.00	0.00	0.00	0.00	0.00	0.00	**0.07**
BC1161	NP_830947	PrsA2	Peptidylprolylisomerase		0.00	0.00	**0.06**	0.00	0.00	**0.06**	0.00	0.00	**0.12**
BC1641	NP_831419	FglB	Flagellar basalbody rod protein		0.00	0.00	0.00	0.00	0.00	0.00	0.00	**0.20**	0.00
BC1642	NP_831420	FglC	Flagellar basalbody rod protein		0.00	**0.13**	**0.27**	0.00	**0.27**	0.00	0.00	**0.47**	**0.13**
BC1687	NP_831462		Unknown		0.00	0.00	0.00	0.00	0.00	**0.09**	0.00	0.00	**0.09**
BC1901	NP_831673		Unknown		0.00	0.00	0.00	0.00	0.00	0.00	0.00	**0.17**	**0.17**
BC4363	NP_834075		Ferrichrome-binding protein	S	0.00	0.00	0.00	0.00	0.00	**0.06**	0.00	**1.15**	**1.44**
BC4546	NP_834253		Ferrichrome-binding protein	S	0.00	0.00	0.00	0.00	0.00	0.00	0.00	0.00	**0.19**
BC5359	NP_835020		Aminopeptidase Y	S	0.00	0.00	**0.06**	0.00	**0.02**	0.00	0.00	0.00	**0.10**
**GROUP** **C**													
BC1669	NP_831739	LytR3	LytR family transcriptionalregulator		0.00	0.00	**0.08**	0.00	**0.05**	0.00	**0.03**	**0.13**	**0.13**
BC4549	NP_834256	IsdC	Cellsurface protein	S, srtB	0.00	0.00	0.00	0.00	0.00	0.00	**0.04**	**0.19**	**0.27**
BC2473	NP_832233		Beta-lactamase	S	0.00	0.00	0.00	0.00	**0.03**	0.00	**0.06**	**0.06**	**0.03**
BC1893	NP_831666		Scaffold protein		0.00	0.00	0.00	0.00	0.00	0.00	**0.07**	**0.07**	**0.35**
BC3221	NP_832962		Surface protein	S, Fib-alpha	**0.32**	**0.30**	**0.62**	**0.32**	**0.67**	**0.81**	**0.09**	**0.42**	**0.99**
BC1862	NP_831635		Unknown		0.00	0.00	0.00	0.00	0.00	0.00	**0.10**	0.00	0.00
BC1634	NP_831412		UDP-N-acetylenolpyruvoylglucosamine reductase		0,00	0.00	0.00	0.00	0.00	0.00	**0.11**	0.00	0.00
BC2186	NP_831951	FtsK	Celldivision protein	S	**0.05**	**0.09**	**0.09**	**0.05**	**0.14**	**0.14**	**0.14**	**0.45**	**0.23**
BC1660	NP_831437		Soluble lytic murein transglycosylase	S, LT_GEWL	**0.04**	0.00	0.00	**0.04**	0.00	0.00	**0.14**	0.00	0.00
BCp0009	NP_829897		DNA packaging		0.00	**0.08**	**0.08**	0.00	**0.16**	**0.08**	**0.16**	0.00	**0.08**
BCp0018	NP_829906		Unknown		0.00	**0.17**	**0.58**	0.00	**0.21**	**0.41**	**0.38**	**0.07**	**0.14**
BC4548	NP_834255		Cellsurface protein	S	**0.17**	**0.17**	**1.34**	0.00	**0.17**	**1.40**	**0.45**	**3.98**	**3.81**

a Domains that are inherently related to the functional annotation of the proteins are not indicated. S, export signal peptide; SH3, SRC homology 3 domain; srtB, sortase B cell surface sorting signal; Fib-alpha, fibrinogen alpha/beta chain family; LTGEWL, lytic transglycosylase and goose egg-white lysozyme (GEWL) domain.

b NSAF values are given at early growth phase (EE), declining growth phase (LE), and stationary growth phase under aerobiosis, high-ORP anaerobiosis, and low-ORP anaerobiosis.

c Group A, proteins not hitherto annotated (NA); Group B, proteins not detected in EE; Group C, proteins not detected in previous studies.

NA, not annotated.

Bold values are greater than zero.

#### Insights into the core-exoproteome of *B. cereus*

**Figure 2:**
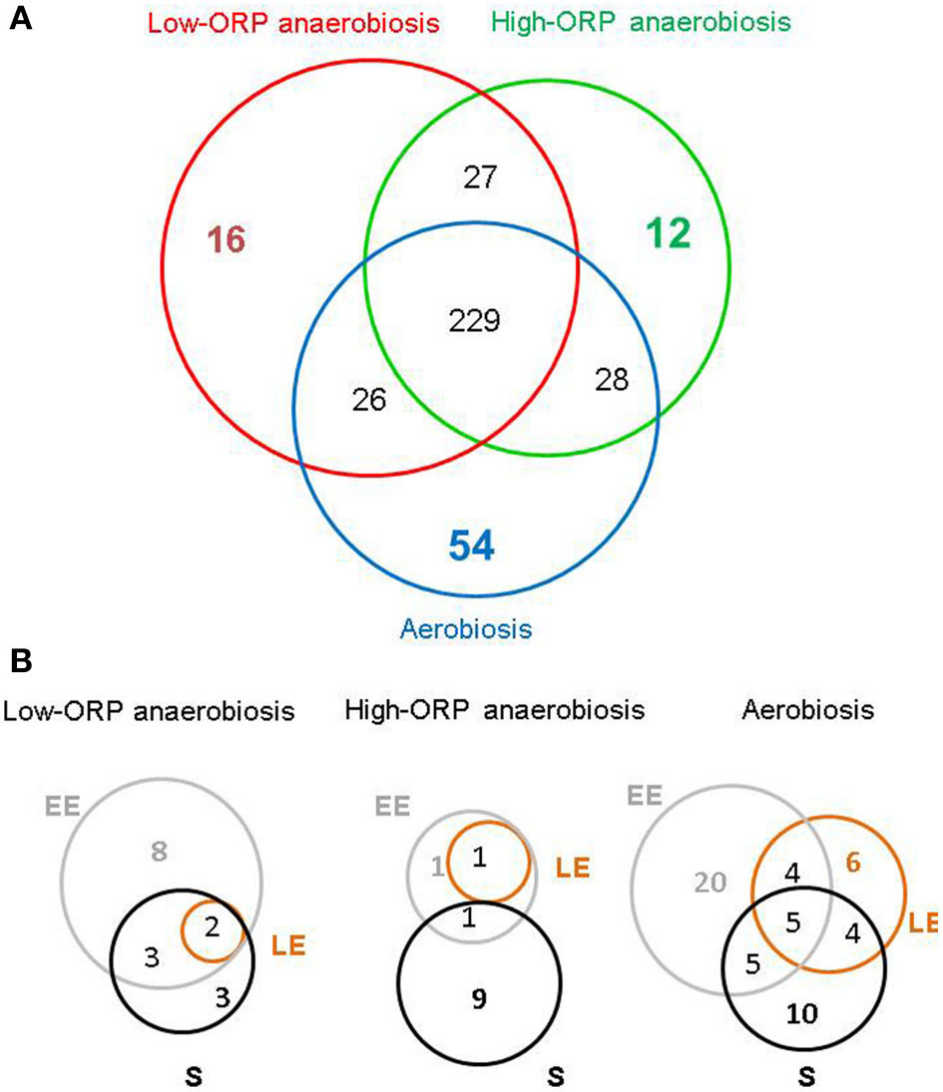
**Venn diagram–based comparison of the exoproteomes identified in the three growth conditions**. **(A)** Number of identified proteins in the exoproteomes obtained from aerobically, and high-ORP and low-ORP anaerobically grown cells. **(B)** Distribution of proteins specifically detected in one growth stage (EE, LE, S) by function of the growth conditions.

Figure 2A, shows a Venn diagram comparing the exoproteomes identified in the three different growth conditions. In this case, 229 of the 392 proteins identified were found to accumulate in the extracellular milieu, whatever the redox growth conditions. Regarding this feature from a quantitative perspective, this core proteome accounts for 89% of the total NSAF. Besides this core exoproteome, 54, 12, and 16 proteins were found exclusively in aerobically, high-ORP- and low-ORP–anaerobically grown cells, respectively. Globally, these proteins are poorly abundant, explaining why some of them were detected in the EE growth phase and not in the LE and S growth phases, especially under aerobiosis (20/54) and low-ORP anaerobiosis (8/16), as shown in Figure 2B. However, 5 and 2 proteins may be considered as fully representative of oxic and low-ORP anoxic conditions, respectively, because they were systematically detected in the three growth phases. The five aerobiosis-specific proteins are: the β-subunit of pyruvate dehydrogenase E1 (PdhB; BC3972), which catalyzes the decarboxylation of pyruvate into acetyl-CoA in oxic conditions; a ribosomal protein (RpsH, BC0145); a putative cell-surface protein (BC4549); a scaffold protein (BC1893); and a putative ferrichrome ABC transporter substrate-binding protein (BC5380). The two proteins that specifically accumulated under low-ORP anaerobiosis are a putative D-3-phosphoglycerate dehydrogenase (BC3248) and a putative nucleoside-binding protein (BC3791). No protein was found to be specifically assigned to high-ORP anoxic conditions.

#### Functional insights into the pan-exoproteome of *B. cereus*

**Figure 3:**
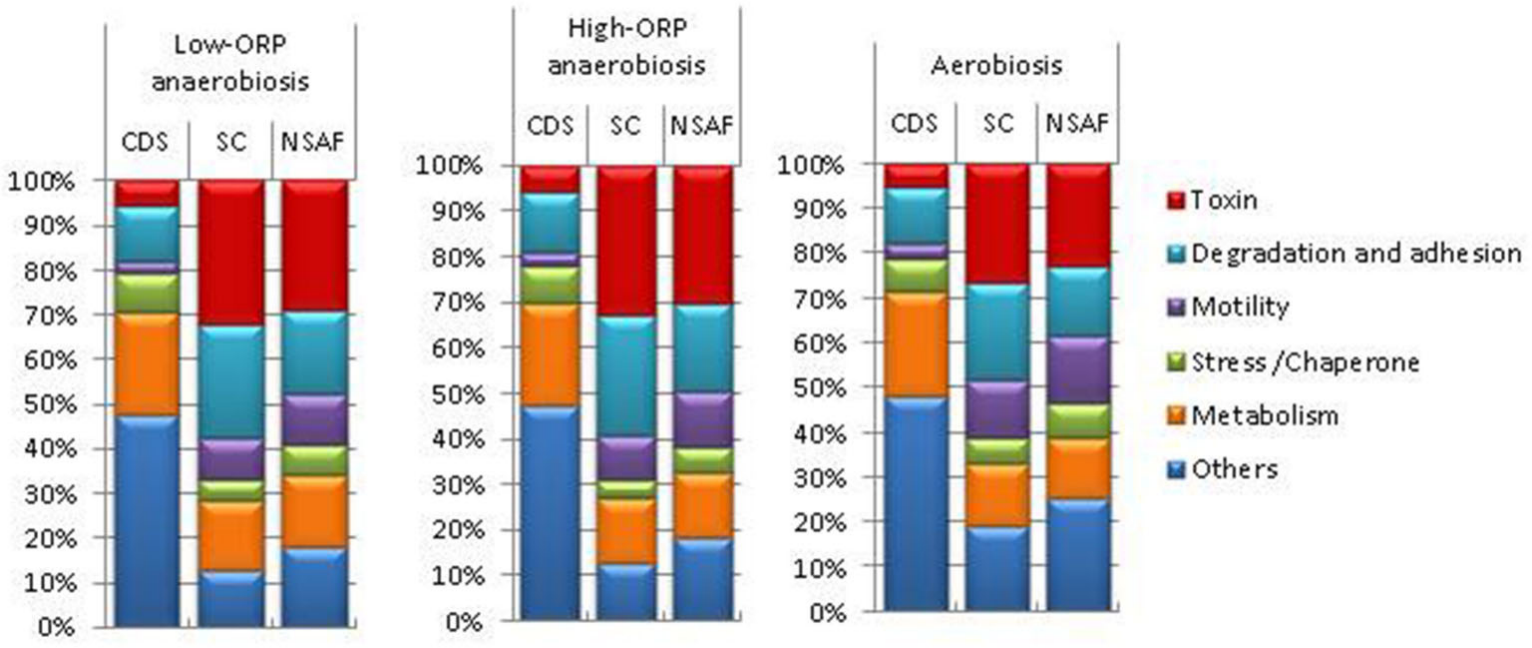
**Relative abundance of MS/MS-detected polypeptides under aerobiosis, high-ORP anaerobiosis, and low-ORP anaerobiosis**. Ratio expressed in percentage of the total numbers of polypeptides (CDS), spectral counts (SC), and protein abundance normalized by the corresponding molecular weight (NSAF) per functional category are represented graphically by stacked bars for each condition.

Figure 3 shows the whole set of exoproteins that were detected for the three growth phases in each growth condition and were classified into six main functional categories. The group “Others” comprises non-classical secreted proteins (translation, transcription, cell division, rod shape–related proteins), extracellular component of transport systems, proteins that are usually anchored to the bacterial membrane, and proteins with no function yet identified. Remarkably, more than 40% of the identified exoproteins (CDS) were classified in this group. Among these, 27 did not show any significant similarities with any known proteins, as determined by BLAST searches against the NCBInr database. Therefore, these could be considered as lineage-specific proteins for the *B. cereus* species (for more details see Table S3 in Supplementary Material). The number of CDS assigned to the toxin-related group is much lower (10-fold) than to the “Others” group, but the toxin-related group was more highly represented in terms of spectral counts (SC) and NSAF, and thus abundant whatever the condition. Toxin-related group represented the largest ratio of the MS/MS-detected peptides, with a range from 26 to 33%. Like the toxin-related group, the motility and stress/chaperone-related groups contain a low number of proteins. However, these two groups represent a lower abundance fraction of the exoproteome than the toxin-related group in the three conditions. Flagella components, usually anchored to the membrane, are the main contributors to the motility group (Table S3 in Supplementary Material). Their presence in the exoproteome could be explained by their fragility. When shaking the culture or removing cells by filtration or centrifugation, they can be easily broken into small pieces. Like the flagella components, the proteins belonging to the group comprising stress- and chaperone-related proteins (such as catalases, superoxide dismutase, GroEL, Dnak, etc.) did not comprise any typical peptide signal. However, they are known as typical components of the exoproteome of pathogens (Armengaud et al., 2012). Adhesion and degradative proteins belong to an abundant fraction of the *B. cereus* exoproteome in the three conditions. The number of proteins dedicated to adhesion functions was lower than those assigned to degradation and the adhesion-related group was also less detected in terms of SC (Table S3 in Supplementary Material). The metabolism group comprises proteins related to central, amino acid, lipid, and fatty acid metabolism. The former subgroup is the most abundant and the latter the least abundant in terms of spectral counts (Table S3 in Supplementary Material). Specifically, Figure 3 shows that the percentages of proteins belonging to the stress/chaperone-related and motility-related groups were higher under aerobiosis than under anaerobiosis, especially under high-ORP anaerobiosis. In contrast, the percentages of toxin-, degradative- and adhesion-related proteins were higher under anaerobiosis than under aerobiosis. The genes/operons involved in flagellum biosynthesis, enzymatic defenses against stress, and virulence factors are known to be tightly regulated in response to the presence or absence of dioxygen (Evans et al., 2011). This may contribute to the changes observed in the exoproteome.

### Principal component analysis of *B. cereus* exoproteome dynamics

PCA was carried out to simplify the exoproteome time-course data of *B. cereus* (Ivosev et al., 2008; Jayapal et al., 2008), following a previous procedure (Clair et al., 2013). We chose to exclude from the original datasets (259 proteins, Table S2 in Supplementary Material) the proteins found in less than two out of the three replicates for each growth phase sample in each condition. Considering the three growth phase–related observations (EE, LE, and S) and the three biological replicates for each observation, datasets for PCA comprised 9 readouts for 88 proteins under low-ORP anaerobiosis, 106 proteins under high-ORP anaerobiosis, and 114 proteins under aerobiosis. These datasets and analytical details are given in Table S4 in Supplementary Material.

#### Overview of exoproteome dynamics

**Figure 4:**
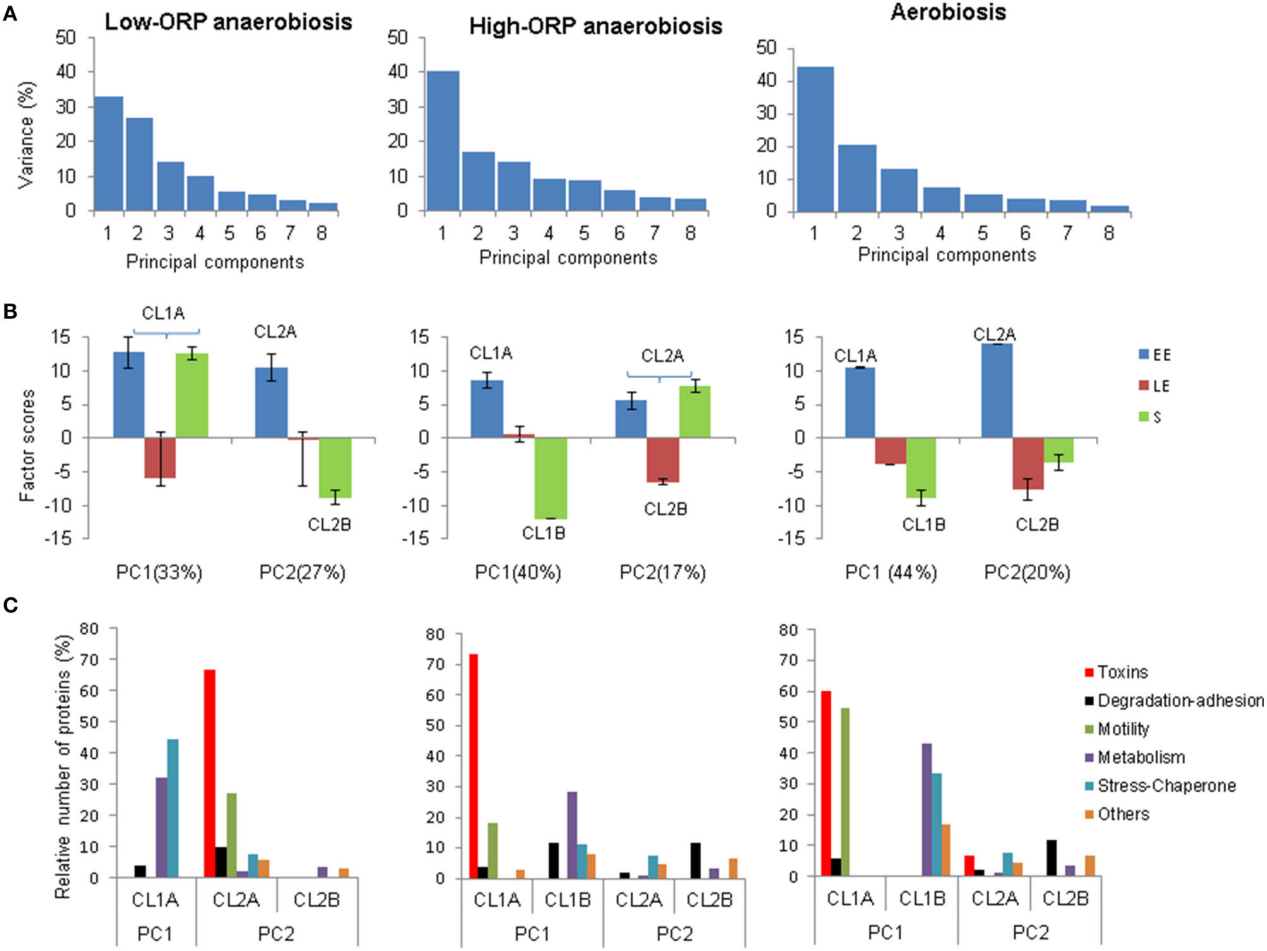
**Principal component analysis of the *B. cereus* exoproteome**. **(A)** Fractions of the variances borne by axes 1–8. **(B)** Growth phase contributions to the first two principal components (PC1 and PC2), under low-ORP anaerobiosis, high-ORP anaerobiosis, and aerobiosis. Protein clusters assigned to growth phases were indicated by (i) the same capital letter **(A)** when they did not show abundance level change in these growth phases, or (ii) different capital letters **(A,B)** when the proteins showed negative correlation with abundance level changes. **(C)** Relative number of proteins assigned to toxins, degradation/adhesion, motility, metabolism, stress/chaperone, and “others” functional groups in protein clusters determined by PCA. Each functional group is represented by a color.

PCAs extracted two principal components (PC1 and PC2), which explained ~60% of the total variance in the three conditions (Figure 4A). Scores and loadings of PC1 and PC2 are different in the three growth conditions (Figure 4B). This indicates that PCA extracted two time-course clusters (represented by PC1 and PC2) that did not contribute equally to the dynamics of the exoproteome in each condition. Figure 4B shows that, under low-ORP anaerobiosis, PC1 represented the tendency of some proteins (co-clustered in CL1A) to be similarly abundant in the EE and S growth phases. PC2 negatively correlates the abundance level decrease of some proteins (CL2A) between the EE and S growth phases with the abundance level increase of other proteins (CL2B). Under high-ORP anaerobiosis PC1 showed the same features as PC2 under low-ORP anaerobiosis and identified two protein clusters, named CL1A and CL1B. PC2 negatively correlates the absence of abundance level change of some proteins (CL2A) between the EE and S growth phases with the abundance level decrease of some proteins (CL2A) between the EE and LE growth phases. Under aerobiosis, PC1 represented the same features as PC1 and PC2 under high- and low-ORP anaerobiosis, respectively and identified two clusters of proteins CL1A and CL1B. PC2 negatively correlates the decrease in abundance level of some proteins (CL2A) with the increase in abundance level of other proteins (CL2B) between the EE and S growth phases.

#### Distribution of functional groups inside kinetic clusters of proteins

All proteins contributing to the CL clusters extracted from PC1 and PC2 were assigned to one of the six functionally distinguished groups established in Figure 3. Figure 4C shows that, under low-ORP anaerobiosis, stress/chaperone- and metabolism-related proteins preferentially contributed to CL1A and toxin- and motility-related proteins to CL2A. Under both high-ORP anaerobiosis and aerobiosis, toxin-, motility-, metabolism-, and stress/chaperone-related proteins preferentially contributed to CL1A. However, CL1A co-clustered a higher number of toxin-related proteins under high-ORP anaerobiosis while it clustered a higher number of motility-, metabolism-, and stress-related proteins under aerobiosis. Taken together, the results show that toxin-related proteins displayed the highest functional-group homogeneity compared to other functionally related proteins in the three growth conditions. Specifically, PCA revealed that the decrease in abundance level of the majority of toxin-related proteins between EE and S growth phases was (i) uncorrelated with the change in abundance level of the majority of metabolism- and stress-related proteins under low-ORP anaerobiosis, (ii) negatively correlated with the increase in abundance level of less than ~30% of metabolism-related proteins under high-ORP anaerobiosis, and (iii) negatively correlated with the increase in abundance level of more than 40 and 30% of metabolism- and stress-related proteins, respectively, under aerobiosis. Studies of metabolic network structures have shown that connected functional groups of proteins may contribute to a common cellular process (Ravasz et al., 2002). Our data raise the question of the role of toxins in *B. cereus* active growth, i.e., in primary metabolism and possibly in cellular protection against metabolism-related oxidative stress in respiring aerobic cells.

#### Focus on the dynamics of toxin-related proteins

Table 2 lists the toxin-related proteins that contributed to CL2A under low-ORP anaerobiosis and CL1A under high-ORP anaerobiosis and aerobiosis. The data show that the three hemolysin BL (Hbl) components (HblL1, HblL2, and HblB) co-clustered with HblB', which is encoded by the *hblB* gene located downstream of the *hblCDA* operon (Clair et al., 2010), in the three conditions. Co-clustering was also observed for the three non-hemolytic enterotoxin (Nhe) components, which are encoded by the *nheABC* operon (Lindback et al., 2004). Hbl and Nhe components also co-clustered with (i) hemolysin II (HlyII) under aerobiosis, (ii) EntB under both aerobiosis and low-ORP anaerobiosis, (iii) EntA and EntC under high-ORP anaerobiosis, and (iv) cytotoxin K (CytK) and Hly I under both high- and low-ORP anaerobiosis. In conclusion, Hbl and Nhe components may constitute the core of the toxin-related clusters and the other proteins constitute the growth condition variance with (i) HlyII representative of aerobic respiratory condition, (ii) CytK and HlyI representatives of the anaerobic fermentative conditions, (iii) EntA and EntC representatives of classical anoxic conditions (high-ORP anaerobiosis), and (iv) EntB representative of both aerobic respiration and low-ORP anaerobic fermentation. These two latter conditions generate endogenous oxidative stress, which is counteracted by antioxidant systems. Among these, OhrRA was found to regulate EntB (Clair et al., 2012). Consequently, EntB could be a marker of oxidative stress–generating conditions.

**Table 2:**
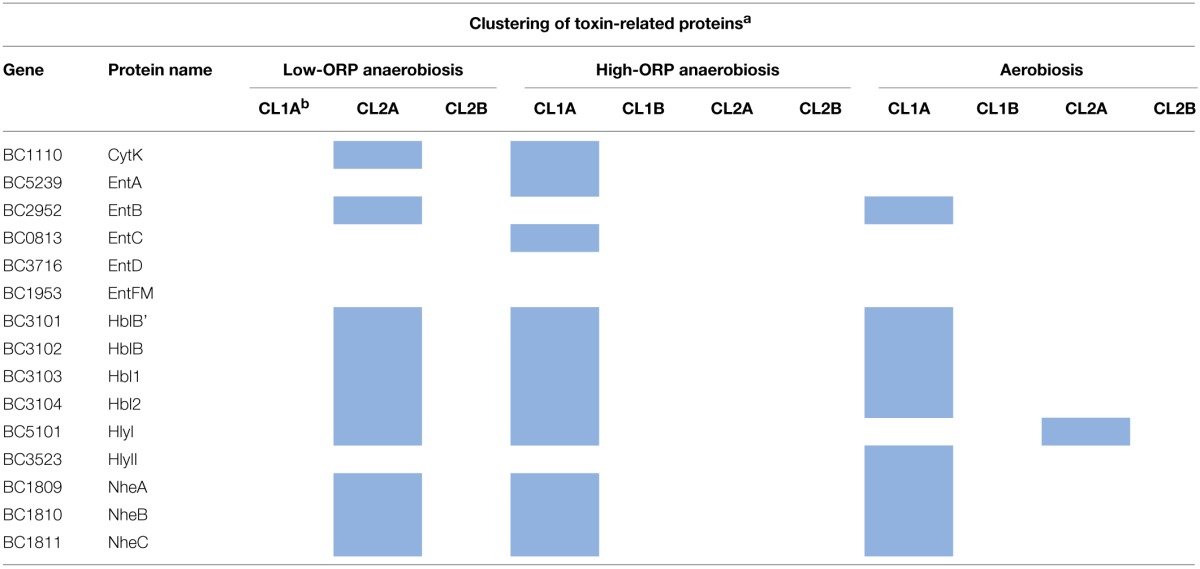
**Clustering of toxin-related proteins during *B. cereus* growth under low- and high-ORP anaerobiosis and aerobiosis**.

^a^Background colors identify proteins that are co-clustered.

^b^Clusters extracted from PCA and contributing to PC1 and PC2 were indicated as CL1 and CL2. The capital letters indicate sub-clusters of CL1 and CL2.

### Dynamics of the Met(O) content of the *B. cereus* exoproteome

In all Gram-positive bacteria, the majority of extracellular proteins need to remain unfolded to be translocated across the plasma membrane, the plasma membrane being known to support the highest level of ROS production in the cell (Fisher, 2009; Schneewind and Missiakas, 2014). On the other hand, Met residues in polypeptidic chains are more sensitive to oxidation than Met residues in mature proteins, as Met residues are usually located in the hydrophobic core of proteins (Fliss et al., 1983; Drazic and Winter, 2014). For these reasons, intracellular ROS may cause significant oxidation of exoproteins prior to their translocation. Insofar as Met(O) residues are not reduced back to Met, and there is no ROS source in the extracellular medium, the Met(O) content of the exoproteome might directly reflect endogenous ROS oxidation. To test this hypothesis, we used nanoLC-MS/MS to assess Met(O) content in all the proteins identified in the exoproteome. We analyzed their time-course dynamics in aerobically grown cells and in anaerobically grown cells for this specific parameter.

#### Overview of methionine oxidation

**Figure 5:**
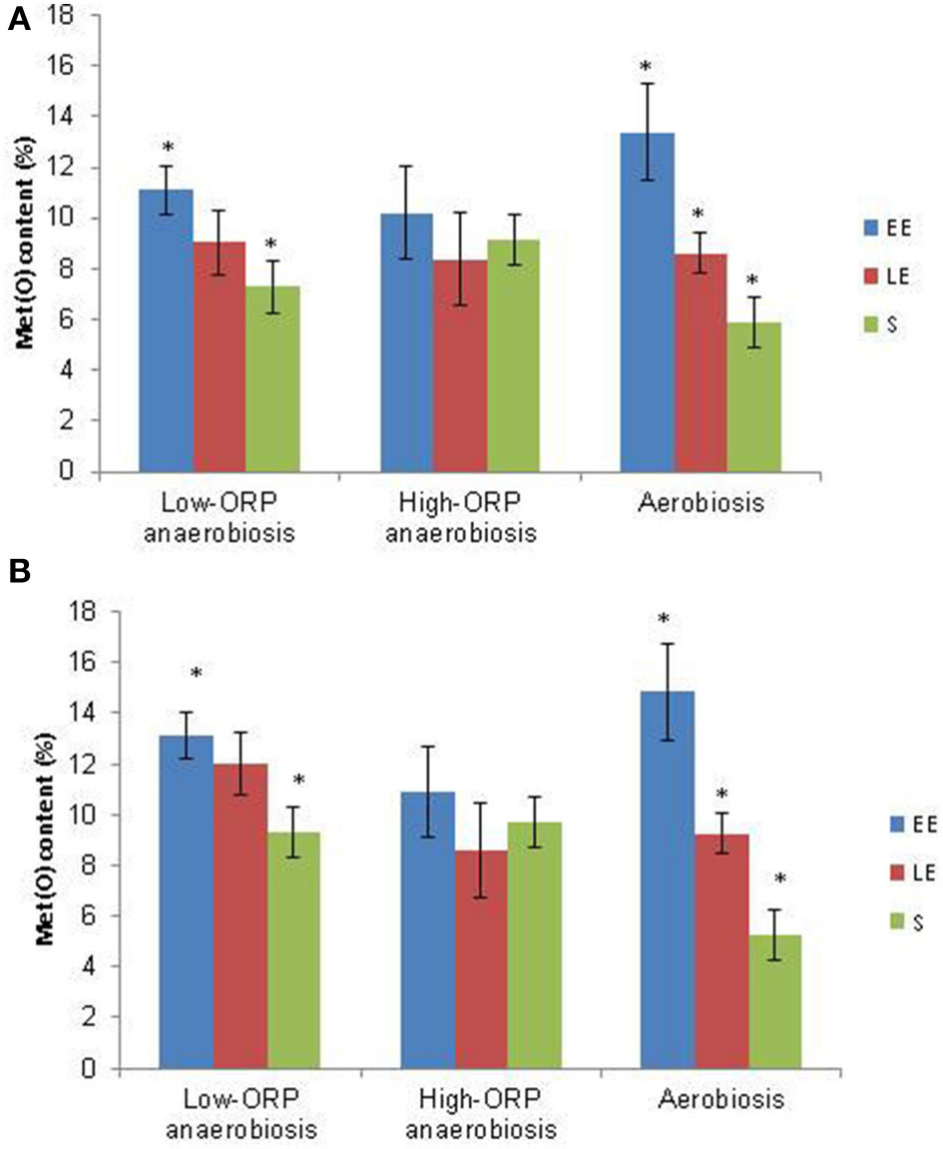
**Dynamics of exoproteome Met(O) content under low-ORP anaerobiosis, high-ORP anaerobiosis, and aerobiosis. (A)** The Met(O) content was calculated as the percentage of the number of all detected Met(O) peptides vs. the total number of MS/MS spectra. **(B)** Only the peptides assigned to proteins that co-clustered in CLM1 (Table S6) were considered. Data are the means of triplicate measures obtained from three independent cultures in each growth condition at the EE, LE, and S growth phases. Significant differences (*p* < 0.05 in Student's *t*-test) between two growth phases are indicated with asterisks.

A total of 4532 peptides containing oxidized Met residue(s) (Met(O) peptides) were identified along the 27 nanoLC-MS/MS runs (Table S1 in Supplementary Material). A total of 211 different Met(O) peptides were listed (Table S5 in Supplementary Material), a significant number of them being detected reproducibly. The Met(O) peptide content of the *B. cereus* exoproteome was estimated as a percentage of the total number of peptides identified in each of the three biological samples obtained for each growth phase sample under low- and high-ORP anaerobiosis and aerobiosis. Figure 5A shows that the Met(O) peptide content of the *B. cereus* exoproteome decreased significantly during growth under low-ORP anaerobiosis and aerobiosis, to reach its minimum in the stationary phase. However, aerobiosis sustains a higher decrease along this kinetic compared to low-ORP anaerobiosis. Strikingly, no significant change was observed under high-ORP anaerobiosis. Similar results were obtained by comparing the number of Met(O) to the total number of Met (Figure S1 in Supplementary Material). The level of Met oxidation as assessed here is a complex result of the balance between endogenous ROS generation on the one hand and the ability of the cell to repair Met on the other. Oxidized Met can be repaired by antioxidant systems (Drazic and Winter, 2014). Under aerobiosis, the high Met(O) peptide content of the EE exoproteome compared to the S exoproteome could reflect either a surplus of ROS generated by the activity of the respiratory chain (Seaver and Imlay, 2001) or a higher activity of the antioxidant systems in S growth phase (Alamuri and Maier, 2006; Vekaria and Chivukula, 2010). Under anaerobiosis, and in the absence of final electron acceptors for respiratory electron processes, *B. cereus* cells ferment glucose (Zigha et al., 2007). Fermentative pathways do not produce ROS as typical metabolic by-products under classical anaerobic conditions (Landolfo et al., 2008). This may explain why there is no change in the Met(O) peptide content of the *B. cereus* exoproteome during growth under high-ORP anaerobiosis. We reported previously that reductive stress, such as is encountered under low-ORP anaerobiosis, caused intracellular redox imbalance at the EE growth phase, and generated a secondary oxidative stress response (Mols and Abee, 2011; Clair et al., 2013). This could increase the ability of anaerobic cells to repair oxidized Met and explain why S growth phase sustains a lower Met(O) content under low-ORP anaerobiosis than under high-ORP anaerobiosis.

#### Identification of proteins with differential abundance levels and Met(O)-content dynamics

**Figure 6:**
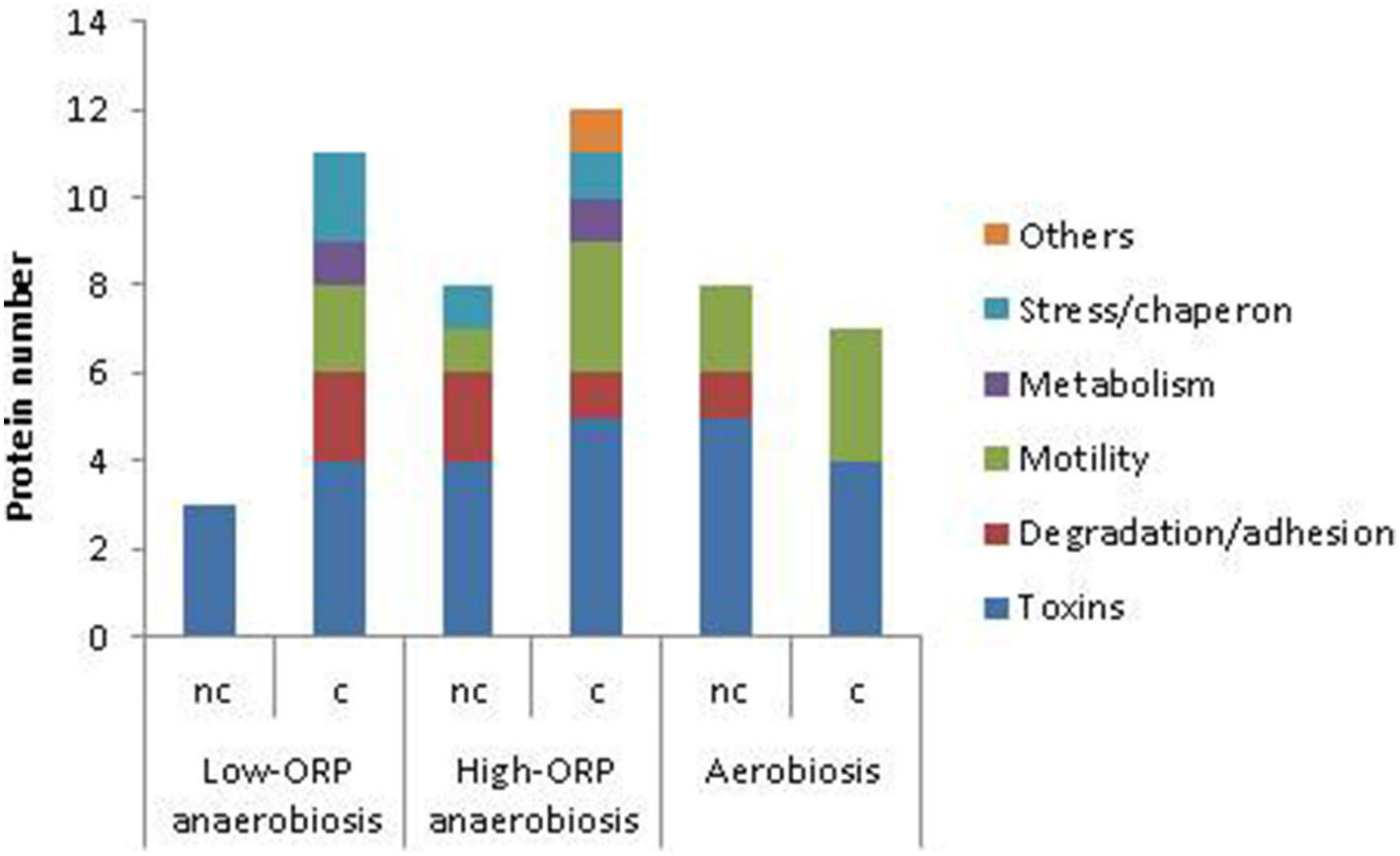
**Characteristics of the protein cluster CLM1 determined by PCA**. Number of proteins assigned to toxins, degradation/adhesion, motility, metabolism, stress/chaperone, and “others” functional groups that co-clustered in CLM1 under low-ORP anaerobiosis, high-ORP anaerobiosis, and aerobiosis. The number of proteins with correlated (c) and uncorrelated (nc) abundance levels and Met(O) content changes is indicated for each growth condition.

**Figure 7:**
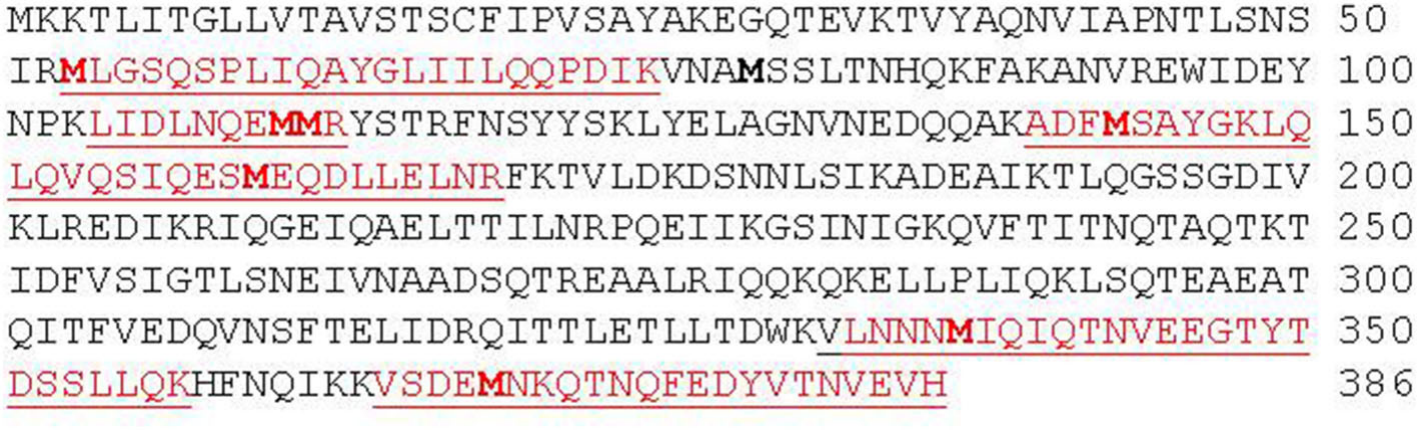
**Amino acid sequence of NheA**. Peptides detected by LC-MS/MS are shown in red and are underlined. Met residues are shown in bold.

To identify proteins exhibiting differences in abundance level and Met(O)-content dynamics, we conducted a second PCA using both abundance (in terms of total number of peptides) and Met(O) peptide content (number of Met(O)-containing peptides) to define proteins in each growth condition. For a robust analysis of the variability in terms of Met(O) peptide content, we considered the proteins containing at least one Met(O) peptide identified in at least two biological replicates. A total of 43 proteins were confidently listed as being oxidized with this criterion (Table S6 in Supplementary Material). Among these, 13 proteins are toxin-related proteins. Remarkably, EntD and HlyII are the only components from the list of detected toxins reported in Table 2 that are not post-translationally modified. The other oxidized proteins are degradative enzymes and adhesins (10), and to a lesser extent, flagella (6), stress-related proteins (4), metabolism-related proteins (7), and uncharacterized proteins (3). PCA extracted 3 Met(O)-related groups (CLM1-3) under low-ORP anaerobiosis, high-ORP anaerobiosis, and aerobiosis (Table S6 in Supplementary Material). Figure 5B shows that CLM1 is representative of the variability of the Met(O) peptide content of the *B. cereus* exoproteome during growth in the three conditions tested. When analyzing the correlation between Met(O) peptide content and abundance level, proteins with differential abundance levels and Met(O)-content dynamics were highlighted. These represent 27, 40, and 53% of proteins co-clustered in CLM1 under low- and high-ORP anaerobiosis, and aerobiosis, respectively (Figure 6). This suggests that oxidation of Met residues may be more specific under aerobiosis than under anaerobiosis. Figure 6 shows that CLM1 comprises a significant subset of Met(O) toxin–related proteins whatever the conditions (7, 9, and 9 under low- and high-ORP anaerobiosis, and aerobiosis, respectively). Table 3 lists the toxin-related proteins that contributed to CML1 and differentiates proteins with similar abundance levels and Met(O)-content dynamics from proteins with differential abundance levels and Met(O)-content dynamics. The data show that HblB, HblL2, HblB', NheA, NheB, and EntB may constitute the core of the toxin-related sub clusters and HblL1, EntA, EntC, and EntFM constitute the growth condition variance with EntFM representative of high-ORP aerobiosis. Table 3 also shows that aerobiosis may sustain higher specific oxidation of Met residues in NheA compared to anaerobiosis. To further strengthen this latter observation, we analyzed the peptides specifically assigned to NheA (Figure 7). Among the 7 Met residues detected in the 6 NheA-assigned peptides reported in Figure 7, four were never detected as oxidized (Table 4 and Supplementary Table S6). This indicates that all NheA-bound methionines are not equally susceptible to oxidation. This may be due to their neighboring amino acids (Ghesquiere et al., 2011). Secondly, NheA contains one Met residue (M53) that is oxidized under anaerobiosis but not under aerobiosis. In addition, NheA contains two adjacent Met residues at positions 111 and 112, which are differentially oxidized under aerobiosis compared to anaerobiosis: oxidation of the first Met residue (M111) occurred only when the second (M112) was oxidized under aerobiosis, while oxidation of M111 did not depend on M112 oxidation under anaerobiosis. Therefore, NheA contains Met residues that respond differently to oxidation under anaerobiosis and aerobiosis. This is also the case for CytK, EntFM, HblB, HblL1, HblL2, and NheC, which all contain one Met residue oxidized under anaerobiosis but not under aerobiosis (Table 5). Thus, anaerobiosis increases the oxidation susceptibility of methionine in toxin-related proteins. This may due to the presence of a different pattern of oxidants in fermentative cells (Mahawar et al., 2012). Taken together, our data indicate that toxin-related proteins contain Met residues that are not equally susceptible to oxidation and Met residue selectivity is a factor that may contribute to Met oxidation under aerobiosis.

**Table 3:**
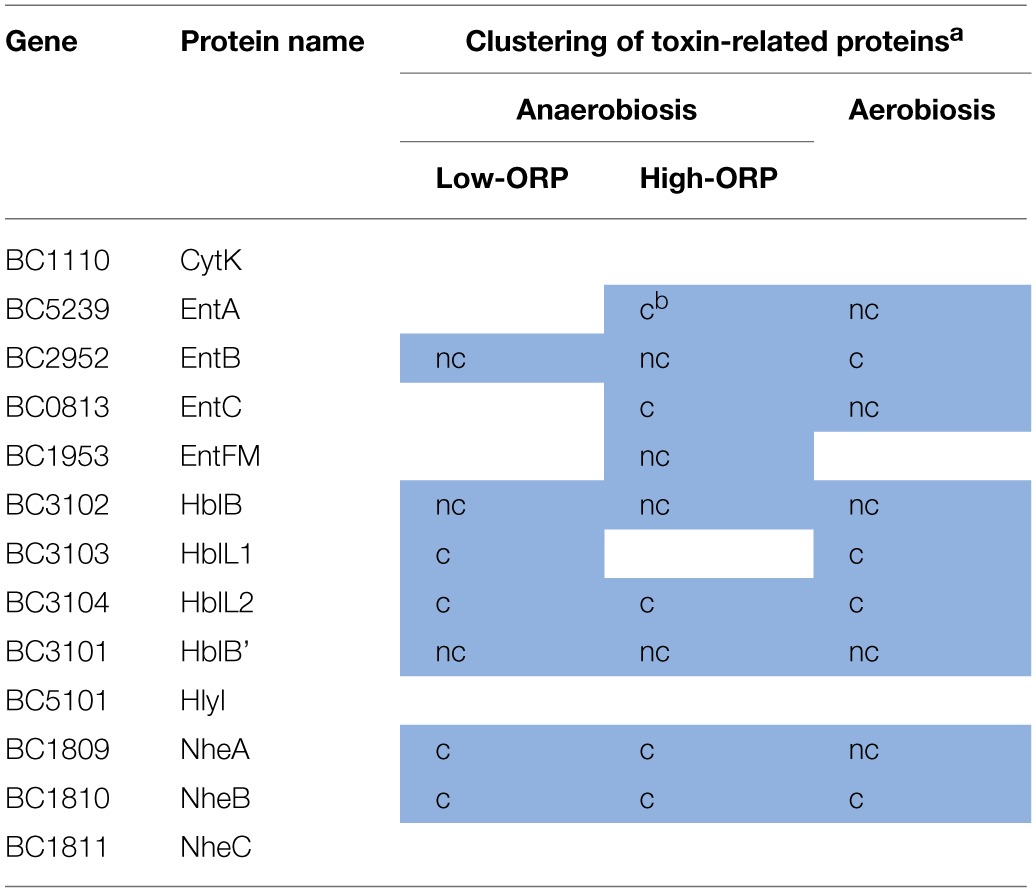
**Co-clustering of toxin-related proteins in CLM1 under low-ORP anaerobiosis, high-ORP anaerobiosis, and aerobiosis**.

^a^Background colors identify proteins that are co-clustered.

^b^The symbols c and nc indicate that the Met(O) peptide content change of a protein is correlated or uncorrelated, respectively, with its abundance level change during growth.

**Table 4:** **List of NheA peptides containing oxidized and non-oxidized Met residues**.

**LC-MS/MS identification**				
**Peptides detected by LC-MS/MS**	**Met^a^**	**Met oxidation**		
		**Anaerobiosis**		**Aerobiosis**
		**Low-ORP**	**High-ORP**	
**M**LGSQSPLIQAYGLIILQQPDIK	M53	M53(O)	M53(O)	nd^b^
		M111(O)	M111(O)	nd
LIDLNQE**MM**R	M111	M111(O) M112(O)	M111(O) M112(O)	M111(O)M112(O)
	M112	M112(O)	M112(O)	M112(O)
ADF**M**SAYGK	M143	nd	Nd	nd
LQLQVQSIQES**M**EQDLLELNR	M160	nd	Nd	nd
VLNNN**M**IQIQTNVEEGTYTDSSLLQK	M337	nd	Nd	nd
VSDE**M**NKQTNQFEDYVTNVEVH	M369	nd	Nd	nd

a Methionine residues (Met) and oxidized Met residues Met(O) were identified by their position in the protein sequence (Figure 7).

b Nd indicates that no oxidized Met residue was detected.

Methionine residues are indicated in bold in peptides detected by LC-MS/MS.

**Table 5:** **Oxidation of Met residues in toxin-related proteins under low-ORP anaerobiosis, high-ORP anaerobiosis, and aerobiosis**.

**Gene**	**Protein name**	**Number of Met residues**		**Number of Met(O) residues**		
		**Total^a^**	**Detected^b^**	**Low-ORP anaerobiosis**	**High-ORP anaerobiosis**	**Aerobiosis**
BC1110	CytK	5	5	1	2	0
BC5239	EntA	3	2	2	2	2
BC2952	EntB	4	2	1	1	1
BC0813	EntC	2	2	2	2	2
BC1953	EntFM	1	1	0	1	0
BC3101	HblB'	13	1	1	1	1
BC3102	HblB	7	6	6	5	4
BC3103	HblL1	7	4	4	4	3
BC3104	HblL2	7	6	6	5	4
BC5101	HlyI	5	2	0	0	2
BC1809	NheA	8	7	3	3	2
BC1810	NheB	4	3	2	2	2
BC1811	NheC	10	2	0	1	0

a The number of Met residues was calculated from the sequence of the mature form of the protein (without peptide signal).

b The numbers reported in this column are the numbers of Met residues detected in our study by LC-MS/MS.

## Conclusion

We used nanoLC-MS/MS data to analyze global changes in the *B. cereus* exoproteome during growth in glucose-containing medium under controlled conditions of pH and pO_2_. We have shown that PCA can identify groups of exoproteins that are coordinately controlled at the growth phase level. The results indicated that proteins belonging to the toxin-related group define characteristic kinetic profiles correlated with the physiological state of the culture in respiring, as in fermenting, cells. The majority of toxin-related proteins accumulated during the exponential growth phase, whatever the conditions. However, their dynamics differ significantly under aerobiosis and anaerobiosis if we consider how their patterns in terms of metabolism, oxidative stress–related proteins and the time dynamics of their Met(O) content are interconnected. Several studies have reported that Met residues of proteins may act as ROS scavengers (Luo and Levine, 2009). It is thus possible that Met residues in toxin-related proteins may act as endogenous antioxidants before being secreted into the extracellular medium. High-level secretion of toxins during the exponential phase may thus contribute to the protection of *B. cereus* cells against cellular oxidation and maintain redox homeostasis by keeping endogenous ROS at bay, especially under aerobiosis. Evidently further studies should be now conducted to confirm these hypotheses. The consequences of methionine oxidation on proteins may vary from structural alterations leading to altered activity and/or altered signal events to protein degradation (Levine et al., 2000). This raises questions about the role of Met oxidation in *B. cereus* virulence, and especially in *B. cereus* cytotoxicity. Indeed, our study demonstrated that the major cytotoxins of the *B. cereus* exoproteome, such as Nhe and Hbl (Sastalla et al., 2013), contain oxidizable methionines, and the effect of oxidation on their biological activity is worthy of documentation.

### Conflict of interest statement

The authors declare that the research was conducted in the absence of any commercial or financial relationships that could be construed as a potential conflict of interest.
