# Supplementary material for: Time dynamics of the Bacillus cereus exoproteome are shaped by cellular oxidation
Source: Front Microbiol. 2015 Apr 22;6:342. doi: 10.3389/fmicb.2015.00342 (PMC4406070; doi:10.3389/fmicb.2015.00342)
Supplement: Supplementary file 1 [file Table1.PDF]

Table S1. Growth parameters from controlled batch cultures of *B. cereus* ATCC 14579[a].

	Anaerobic fermentative growth		Aerobic respiratory growth
	iORP = -390 ± 35 mV	iORP = 130 ± 20 mV	iORP = 210 ± 13 mV
Final ORP (mV)	-410 ± 10	-106 ± 16	180 ± 11
Maximal specific growth rate (μ_{max}) (h^{-1})	1.02 ± 0.08	0.91 ± 0.04	1.66 ± 0.21
Final biomass (g.liter^{-1})	1.23 ± 0.06	1.25 ± 0.07	1.72 ± 0.02
$Y_{glucose}$ (g of cells. mol of glucose^{-1})	41 ± 1	41 ± 2	74 ± 2
Maximal specific glucose consumption(mmol.g^{-1}.h^{-1})	25 ± 2	25 ± 2	22 ± 1
Yields of end products (mol.mol glucose^{-1})[c]			
Lactate ($Y_{lactate}$)	0.42 ± 0.04	0.41 ± 0.05	0.02 ± 0.01
Acetate ($Y_{acetate}$)	0.17 ± 0.03	0.27 ± 0.03	0.56 ± 0.04
Formate ($Y_{formate}$)	0.26 ± 0.02	0.27 ± 0.02	0.011 ± 0.001
Ethanol ($Y_{ethanol}$)	0.11± 0.01	0.08± 0.01	NZ
Succinate ($Y_{succinate}$)	0.01 ± 0.00	0.01 ± 0.00	0.02 ± 0.00
Extracellular proteins (mM)	0.14	0.25	0.39

[a]Cells were grown under H_2 and N_2- generated anaerobiosis (pO_2 =0%) and full aerobiosis (pO_2 =100%). Data are the means of triplicate measures obtained from three independent cultures
[c]Yields of end products were calculated at the stationary phase.
[d]NZ, yield was below 0.01 mol.mol glucose^{-1}.
