# Supplementary figures and images for: Time dynamics of the Bacillus cereus exoproteome are shaped by cellular oxidation
Source: Front Microbiol. 2015 Apr 22;6:342. doi: 10.3389/fmicb.2015.00342 (PMC4406070; doi:10.3389/fmicb.2015.00342)

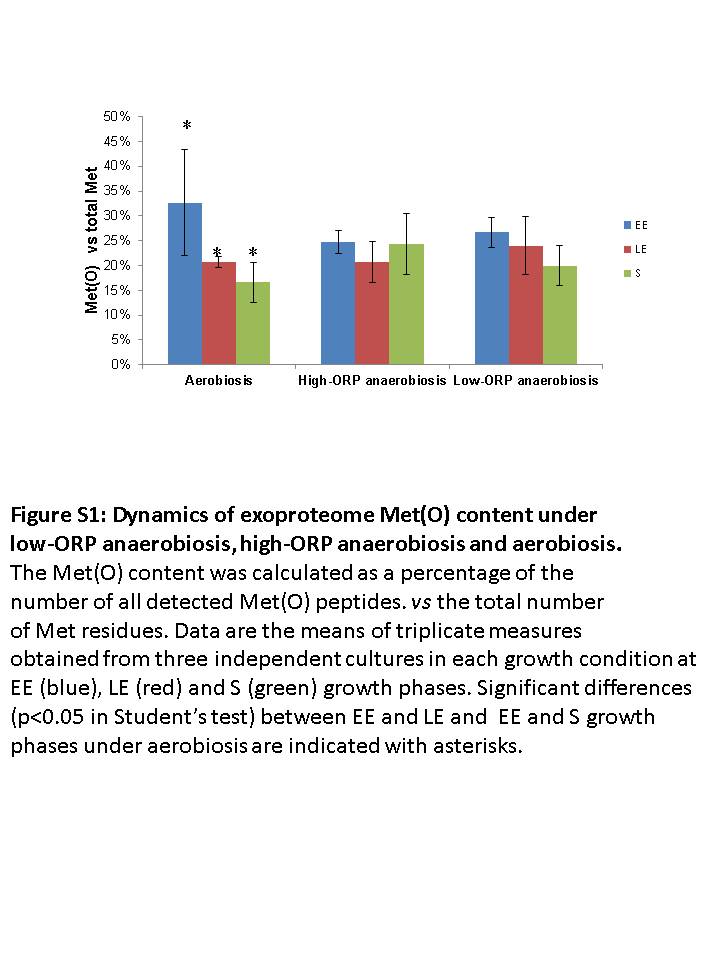

Supplement: Supplementary file 7 [file Image1.JPEG]
